# Targeted panel sequencing in the routine diagnosis of mature T- and NK-cell lymphomas: report of 128 cases from two German reference centers

**DOI:** 10.3389/fonc.2023.1231601

**Published:** 2023-08-16

**Authors:** Julia Böck, Katja Maurus, Elena Gerhard-Hartmann, Stephanie Brändlein, Katrin S. Kurz, German Ott, Ioannis Anagnostopoulos, Andreas Rosenwald, Alberto Zamò

**Affiliations:** ^1^ Institute of Pathology, University of Würzburg, Würzburg, Germany; ^2^ Department of Clinical Pathology, Robert-Bosch-Hospital, and Dr. Margarete Fischer-Bosch Institute of Clinical Pharmacology, Stuttgart, Germany

**Keywords:** T-cell lymphoma, panel-sequencing, NGS, diagnostics, mutation, FFPE

## Abstract

Diagnosing any of the more than 30 types of T-cell lymphomas is considered a challenging task for many pathologists and currently requires morphological expertise as well as the integration of clinical data, immunophenotype, flow cytometry and clonality analyses. Even considering all available information, some margin of doubt might remain using the current diagnostic procedures. In recent times, the genetic landscape of most T-cell lymphomas has been elucidated, showing a number of diagnostically relevant mutations. In addition, recent data indicate that some of these genetic alterations might bear prognostic and predictive value. Extensive genetic analyses, such as whole exome or large panel sequencing are still expensive and time consuming, therefore limiting their application in routine diagnostic. We therefore devoted our effort to develop a lean approach for genetic analysis of T-cell lymphomas, focusing on maximum efficiency rather than exhaustively covering all possible targets. Here we report the results generated with our small amplicon-based panel that could be used routinely on paraffin-embedded and even decalcified samples, on a single sample basis in parallel with other NGS-panels used in our routine diagnostic lab, in a relatively short time and with limited costs. We tested 128 available samples from two German reference centers as part of our routine work up (among which 116 T-cell lymphomas), which is the largest routine diagnostic series reported to date. Our results showed that this assay had a very high rate of technical success (97%) and could detect mutations in the majority (79%) of tested T-cell lymphoma samples.

## 1 Introduction

T- and NK-cell lymphomas are relatively rare and usually aggressive neoplastic diseases, which notoriously pose diagnostic difficulties due to their rarity, but also due to their intrinsic morphological subtlety and partial lack of strongly specific diagnostic markers. A diagnosis of mature T- or NK-cell lymphoma (actually comprising more than 30 diagnostic categories in the 5^th^ edition of WHO classification as well as in the International Consensus Classification - ICC) (1, 2) requires a combination of clinical data, morphological alterations, immunophenotypical features and clonality testing, none of which is singularly diagnostic. The molecular detection of clonal T-cell populations by means of consensus primer PCR amplification and capillary electrophoresis of the TCR gamma (*TCRG*) or beta (*TCRB*) rearranged gene segments has become a determining factor for the diagnosis of difficult cases (3). However, it is commonly negative in NK-derived lymphomas and can be positive in a number of reactive conditions as well as in many normal individuals.

Due to the recently discovered molecular alterations in T-cell lymphomas, targeted next-generation sequencing (NGS) of commonly mutated genes seems an appealing complementary approach for the detection of abnormal T-cell populations (4–6), which might also help in the precise subclassification and provide therapeutically relevant information.

Only few reports describe real-life experience with targeted NGS panels (7–10), and only one focuses specifically on an approach for T-cell lymphomas (10). Focusing on a purely diagnostic approach, we have designed and tested a custom-made targeted NGS panel for T-/NK-cell neoplasias with the following features in mind: a) the mutational pattern should provide support for differential diagnosis within a reference setting as well as answer clinical questions; b) the test should be easily and rapidly implementable within our common NGS routine diagnostic pipeline; c) the panel should be relatively small (≤ 30kb), so that it could be run on a single case basis (i.e. not requiring batch runs), and might allow sequencing in a pool with different NGS panels of other diagnostic cases of the day-to-day routine; d) the test should work on difficult samples (formalin-fixed paraffin-embedded (FFPE) material, low input, decalcified bone marrow samples, poor DNA quality). Here we report on the design and performance of our panel, tested on 128 prospective cases from the clinical routine of two major German reference centers for hematopathology (Würzburg and Stuttgart).

## 2 Materials and methods

### 2.1 Case series

The majority of cases was tested immediately after completion of standard diagnostic procedures (morphology, immunohistochemistry and in a subset of cases clonality studies), in 10 cases the sequencing was performed following a specific clinical request (mutation status of *TET2*, *STAT3*, *STAT5B*, *IDH2*, *DNMT3A* or *RHOA*). DNA was either previously available from clonality testing or newly extracted. Genomic DNA was isolated from FFPE tissue using the Maxwell RSC Blood DNA Kit (Promega GmbH, Walldorf, Germany). Briefly, samples were pre-treated with a THG1-Thioglycerol/incubation buffer mix for 10 minutes at 80°C followed by an incubation-step overnight with proteinase K at 65°C. Extraction was performed on the Maxwell® RSC 48 instrument. DNA was quantified by quantitative PCR using the TaqMan RNase P Detection Reagents Kit (Life Technologies, Darmstadt, Germany).

T-cell receptor rearrangement assays were performed according to the Euroclonality/BIOMED-2 protocol (3). Ethical approval was granted from the local ethical committee for the use of leftover samples. Samples were reviewed from expert hematopathologists to determine tumor-cell content/cell population of interest. Criteria for inclusion were: a) diagnosis of T-cell lymphoma according to the WHO classification (revised 4th edition) or T-cell proliferation suspicious for T-cell lymphoma; b) tumor cell content/cell population of interest ≥ 10% and; c) DNA concentration of ≥ 1ng/µl (with total DNA quantity ≥ 10ng).

In total, we included 128 cases consisting of 60 T-cell lymphomas with T follicular helper (T)FH-cell phenotype (TCL-TFH; including both angioimmunoblastic T-cell lymphomas (AITL) and nodal peripheral T-cell lymphoma with TFH-phenotype (NPTCL-TFH)); 21 peripheral T-cell lymphomas, not otherwise specified (PTCL, NOS); a group of 7 intestinal T-cell neoplasias (ITCN), including 2 enteropathy-associated T-cell lymphomas (EATL) and 3 monomorphic epitheliotropic intestinal T-cell lymphomas (MEITL) as well as 1 intestinal T-cell lymphoma, NOS (ITCL, NOS) and 1 indolent T-cell lymphoma of the gastrointestinal tract (iTCLGIT); a group of 12 anaplastic large-cell lymphomas (ALCL, of those one breast-implant associated (BIA-ALCL), 2 ALK-positive, the remaining ALK-negative); a group of 8 cutaneous T-cell neoplasias (CTCN), including 3 Mycosis fungoides samples (MF), 3 Sezary syndrome samples (SS) and 2 subcutaneous panniculitis-like T-cell lymphomas (SCPL-TCL); 3 extranodal NK/T-cell lymphomas (ENNK/TCL); and a group of 5 other T-cell lymphoma cases (other T); we also included a group of 8 lymphoma cases which were finally classified as B-cell lymphoma (other non-T, thereof 2 classical Hodgkin lymphomas (C-HL), 1 nodular lymphocyte predominant Hodgkin lymphoma (NLPHL), 1 diffuse large B-cell lymphoma, not otherwise specified (DLBCL, NOS), 1 EBV-positive DLBCL and 2 marginal zone lymphomas (MZL)); and a group of 5 cases, which were eventually classified as reactive (including 1 infectious mononucleosis case (IM)).

### 2.2 Multiplex PCR-based panel sequencing

Panel design was conducted with the Ion AmpliSeq Designer software (v7.48). A full list of covered genes with the respective genomic coordinates is shown in **Supplementary Table S1**. Libraries were prepared using the Ion AmpliSeq Library Kit 2.0 according to the manufacturer’s recommendations and quantified via qPCR using the Ion Library TaqMan Quantitation Kit (Life Technologies). Afterwards, templating and enrichment was performed with the Ion OneTouch 2 and Ion OneTouch ES automated systems or the Ion Chef System, followed by sequencing on the Ion GeneStudio S5 Plus System. Data were analyzed using Torrent Suite Software (v5.16) and Ion Reporter Software (v5.18) (Thermo Fisher Scientific, Darmstadt, Germany).

### 2.3 Data evaluation

Analyzed data were filtered for somatic exonic non-synonymous variants, small insertions/deletions and splice site variants in the flanking regions with an allele frequency ≥ 2%. To remove technical artefacts (e.g., PCR artefacts, fixation artefacts), all variants with an allele frequency lower than 10% and a coverage lower than 200x were visualized by Integrative Genomics Viewer (v2.15.4) (11). Variants with a minor allele frequency of ≥ 0.2% in the general population listed in dbSNP database, ExAC database and gnomAD database were also excluded from further analysis. Variants were classified in likely oncogenic and oncogenic variants or variants of unknown significance (VUS) using the current literature and various databases including cBioPortal, ClinVar, OncoKB, CKB and COSMIC. Nonsense and frameshift mutations in tumor suppressor genes, that were not molecularly characterized yet, were classified as loss of function mutations and therefore as likely oncogenic variants. Results are shown in **Supplementary Table S2**.

## 3 Results

### 3.1 Data quality

**Figure 1:**
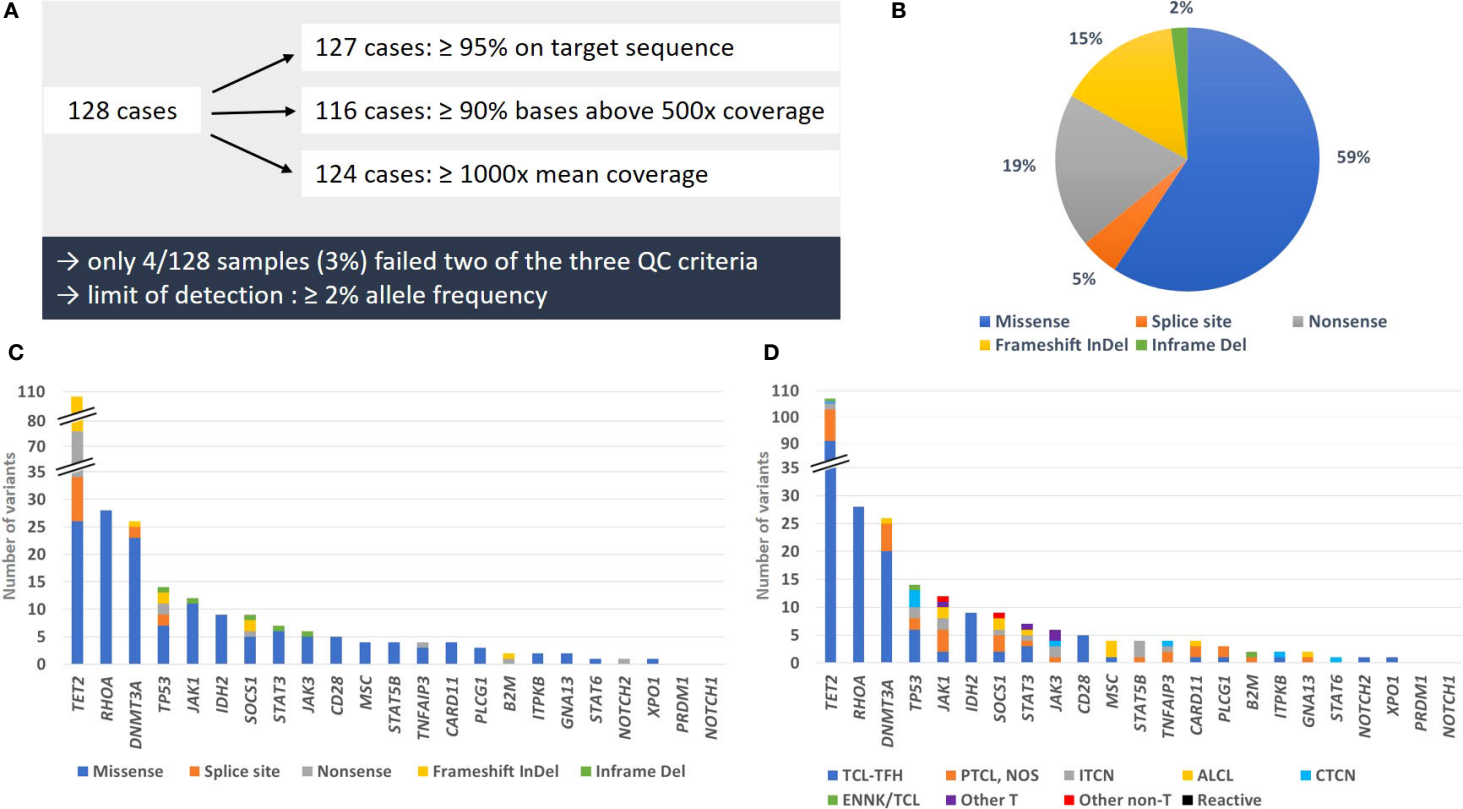
Summary of quality parameters, types of mutations and mutation frequency. **(A)** Overview of cases matching the different QC criteria. **(B)** Proportion of mutation types detected in the studied cohort. **(C)** Number of genetic alterations per mutation type for each analyzed gene. **(D)** Number of variants per T-cell lymphoma subtype and control groups for each analyzed gene. All likely oncogenic/oncogenic variants and variants of unknown significance we detected in this study were counted for illustrations **(B–D)**.

We have analyzed a cohort of 128 cases from two reference centers, comprising 116 T-cell lymphoma samples and 12 non T-cell lymphoma samples. After DNA quantification, all 128 samples were deemed appropriate for further processing. Quantity of all prepared libraries was sufficient for sequencing. Over all samples mean coverage was 4856x, mean of bases above 500 reads was 94% and mean of reads on target was 99% (**Supplementary Table S4**). Defining a threshold for quality control after sequencing, 99% (127/128) of cases showed ≥ 95% reads on target sequence, 91% (116/128) of cases ≥ 90% bases with a coverage above 500 reads and 97% (124/128) of cases a mean coverage of ≥ 1000 reads (**Figure 1A**). Only four samples (3%) failed two of these three quality control criteria. However, in three of those cases mutations with a limit of detection of ≥ 2% allele frequency were reliably detectable, so that the overall quality was individually and finally classified as sufficient.

### 3.2 Mutational data

**Figure 2:**
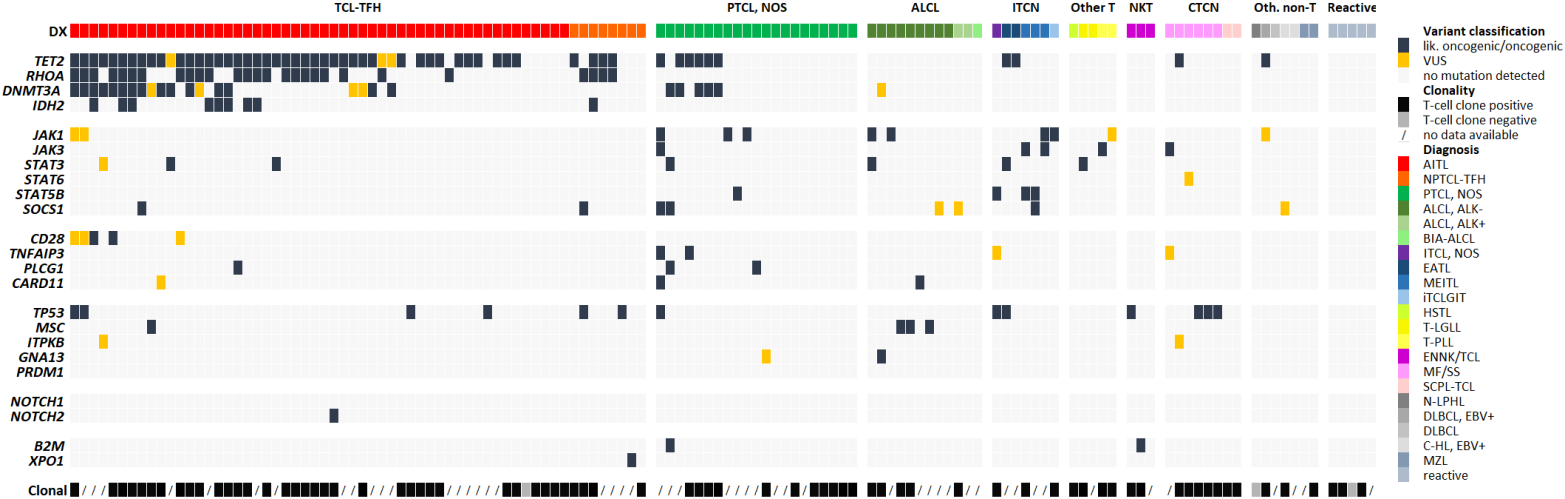
Genetic alterations detected via NGS-based panel sequencing and clonality results. Each row represents one of the genes included in the panel and each column one analyzed sample. If several variants were present in a gene, the most damaging mutation is illustrated and counted as one alteration. Cases are sorted by diagnosis and within the group per frequency of altered genes. Available clonality results are shown in the last row.

Considering the whole collective, we detected 249 somatic mutations, which could be classified as likely oncogenic and oncogenic variants or VUS. Counting all variants per gene as one alteration, we found in 40 samples (31%) only one altered gene, whereas 29 (23%) showed two, 16 (13%) three, 5 (4%) four, 2 (2%) five, 2 (2%) six and 1 (1%) seven altered genes. Overall, 95/128 of the total tested samples (74%) and 92/116 T-cell lymphoma samples (79%) had at least one relevant mutation (**Figure 2**). In 23 out of 116 T-cell lymphoma samples (20%), we could not detect any genetic alteration.

The most frequently mutated genes were *TET2* (58/128 total cases, 45%; and 57/116 T-cell lymphoma cases, 49%), *RHOA* (24/116, 21%), *DNMT3A* (20/116, 17%), *TP53* (13/116, 11%) and *JAK1* (11/128 total cases, 9%; and 10/116 T-cell lymphoma cases, 9%) (**Figure 1C**). All affected genes were exclusively mutated in T-cell lymphoma, except for *TET2* and *JAK1*. The largest proportion of mutations were missense mutations (59%), followed by nonsense mutations (19%), frameshift mutations (15%), splice site mutations (5%) and inframe deletions (2%) (**Figure 1B**). Excluding VUS, the overall picture changed only slightly, with missense mutations now constituting 54% of all variants, followed by nonsense mutations (22%), frameshift mutations (17%), splice site mutations (6%) and inframe deletions (1%) (**Supplementary Tables S2, S3**). Solely missense mutations were detected in *RHOA*, hotspot p.G17V and in *IDH2*, hotspot p.R172K/G/S, typical for AITL, and in *MSC*, hotspot p.E116K, typical for ALCL with DUSP22-translocation (**Supplementary Table S2**). The tumor suppressor genes *TET2*, *TP53* and *SOCS1* showed in addition to missense mutations also different types of truncating mutations, including frameshift, nonsense and splice site mutations (**Figure 1C**). All other genes displayed mostly missense mutations.

Of all detected mutations, 85% (212/249) were classified as likely oncogenic and oncogenic variants and only 15% (37/249) as VUS. About one third of all functionally relevant mutations were truncating mutations in tumor suppressor genes (95/212, 45%) or classical hotspot mutations (40/212, 19%). VUS were detected in 12/23 genes, most frequently in *TET*2 (11/107 variants), *DNMTA3* (6/25 variants) and *JAK1* (5/12 variants). They were almost exclusively missense mutations, with four exceptions in *JAK1*, *JAK3*, *DNMT3A* and *SOCS1*, which had inframe deletions and one frameshift mutation (**Supplementary Table S2**).

Data evaluation showed that the panel is very well suited for diagnostics of T-cell lymphomas with TFH phenotype (AITL and nodal PTCL with TFH-phenotype, currently under the common umbrella of TFH-cell lymphomas both in the 5^th^ edition of the WHO classification and in the ICC) (1, 2), with 48/53 (91%) cases showing at least one mutation, most frequently of *TET2* (45/53, 85%), *RHOA* (23/53, 47%), *DNMT3A* (19/53, 36%) and *IDH2* (8/53, 15%) (**Figure 1D**). In PTCL, NOS the detection rate was only moderate, with 12/21 cases (57%) showing at least one mutation, whereas performance of the panel was high for ALCL (overall 9/12 positive; 75%). Additionally, we obtained a high detection rate for MEITL (3/3 cases), EATL (2/2 cases) and MF/SS (6/6 cases), but the number of tested patients was too low to draw a definitive conclusion (**Figure 2**).

### 3.3 Clonality analysis

Data about clonally rearranged T-cell receptor genes was available in 78 of 128 (61%) cases from routine testing. We also correlated our sequencing results with standard clonality detection (**Figure 2**; **Supplementary Table S5**). Clonally rearranged T-cell receptor genes were detected in 75 out of 78 (96%) tested samples. Of these positive samples, 57 (76%) had at least one mutation, while no genetic alteration was detected in the negative cases. However, 18 cases (24%) with no mutation showed rearranged T-cell-receptor gene fragments. 38 of the 50 non-tested samples (75%) comprised at least one mutation whereas 12 cases (24%) showed no genetic alteration at all. However, the mutation detection rate in rearranged positive samples and non-tested cases was nearly identical (76% compared to 75%).

## 4 Discussion

Mature T-cell lymphomas include more than 30 entities in the 5^th^ edition of the WHO classification as well as in the ICC (1, 2), with the most frequent group AITL (now denominated nodal TFH-cell lymphoma, angioimmunoblastic type or TFH- lymphoma, angioimmunoblastic type) comprising about 1-2% of total lymphoma diagnoses. Practically all T-/NK-cell lymphomas are therefore rare entities, a fact that, in addition to a sometimes challenging morphology, poses diagnostic difficulties in the differential diagnosis with reactive/benign processes as well as with B-cell lymphomas or lymphoproliferations, and occasionally also with Hodgkin lymphomas. A diagnosis of T-/NK-cell lymphoma requires the integration of clinical data, morphology, immunophenotype and molecular information, with no single element *per se* being completely diagnostic. T-cell clonality detection through multiplex PCR of the rearranged T-cell-receptor gene fragments has proven a valuable tool to facilitate a diagnostic decision that usually bears heavy therapeutic consequences. The availability of a nearly complete genetic landscape of mature T-cell lymphomas (12–18) has recently allowed a mutation-based approach to diagnosing these malignancies. The first two notable reports in this sense are the results of a Swiss group (9) and an American group (8), which independently reported their routine experience with the application of targeted panel sequencing in lymphoma diagnostics. Pillonel et al. reported their experience with 80 highly selected, mixed lymphoma cases (including 17 T- and NK-cell neoplasias) collected over three years. Panel sequencing was requested either by the treating physician or by the reference pathologist. Their custom panel included 68 genes and they used Thermo Fisher technology (S5 system) with a sequencing success rate of 98% (78/80 samples with complete panel coverage) (9). The approach by Davis et al. was diametrically opposite, with a large-scale approach sequencing of 518 patients/598 samples (corresponding to 55% of all lymphoma diagnoses during one year) using a commercially available targeted panel (Illumina TruSight Lymphoma). The panel contained 40 lymphoma-related genes and was run on an Illumina MiSeq instrument; the authors report a sequencing success (defined as the detection of a disease-associated variant, of a variant of unknown significance or no variants) of 95% for intramural cases and 77% for extramural cases. Their series included 40 T-cell neoplasms (miscellaneous) where they identified 18 disease-associated variants and 8 variants of unknown significance (8).

More recently, a French group reported on their experience in routine use of a custom capture-based T-cell-oriented panel including 69 genes, which was sequenced using Illumina technology (MiseqDX or NextSeq 550) (10). In contrast to the first two papers mentioned, the authors focused on T-cell lymphomas (82 cases) and reactive T-cell infiltrates (25 cases), comparing the results of panel sequencing with conventional clonality analysis. They detected mutations in 79/82 (96%) T-cell lymphomas, including four cases, which were non-amplifiable by conventional PCR, but none in “reactive T-cell infiltrates” (not further specified). However, the latter showed a clonal population in 13/25 (52%) cases, which argues for a higher specificity of panel sequencing in comparison to T-cell clonality studies.

With special focus on T-cell lymphomas, our approach is more comparable with the work of the French group, but we analyzed a larger series of samples in a real-world environment; to our knowledge, we tested the largest diagnostic series of T-cell lymphomas so far (116 cases). Compared to previous publications, we have focused on developing a NGS-based test providing diagnostically relevant and robust information supporting a diagnosis of T-cell neoplasia within a clinically acceptable time-frame and at a relatively low cost. In our opinion, the panel could be easily implemented in most molecular pathology laboratories that are using a similar NGS pipeline. Our panel design proposes an innovative approach, because: a) it assists in answering diagnostically, prognostically or therapeutically relevant questions, based on our experience within the two reference centers; b) it comprises a small number of genes (23, about 29kb), mostly focusing on hotspots and genes mutated with high frequency and non-redundantly in T-/NK-cell lymphomas; c) it follows a mix-and-match approach, with the intent of maximum flexibility, because it can be combined with other diagnostic panels on an everyday basis, also single samples can be run; d) our protocol has a short turn-around-time (ten working days to final report) thanks to the use of a PCR-based protocol and implementation on an Ion S5 system, as well as relatively low costs. In order to optimize the size and efficiency of our panel, we did not take copy number variations (CNV) into consideration.

As detailed in the results section, we had a very high sequencing success rate (97%), despite using samples from different external sources and decalcified samples. This high rate is probably due to the small size of the panel, which provides a high level of coverage for each region and the power of amplicon-based library preparation of samples with poor DNA quality and quantity. In fact, only four samples (3%) failed two of the three stringent quality control criteria, but in three cases relevant mutations were reliably detectable and were therefore deemed as valid.

**Figure 3:**
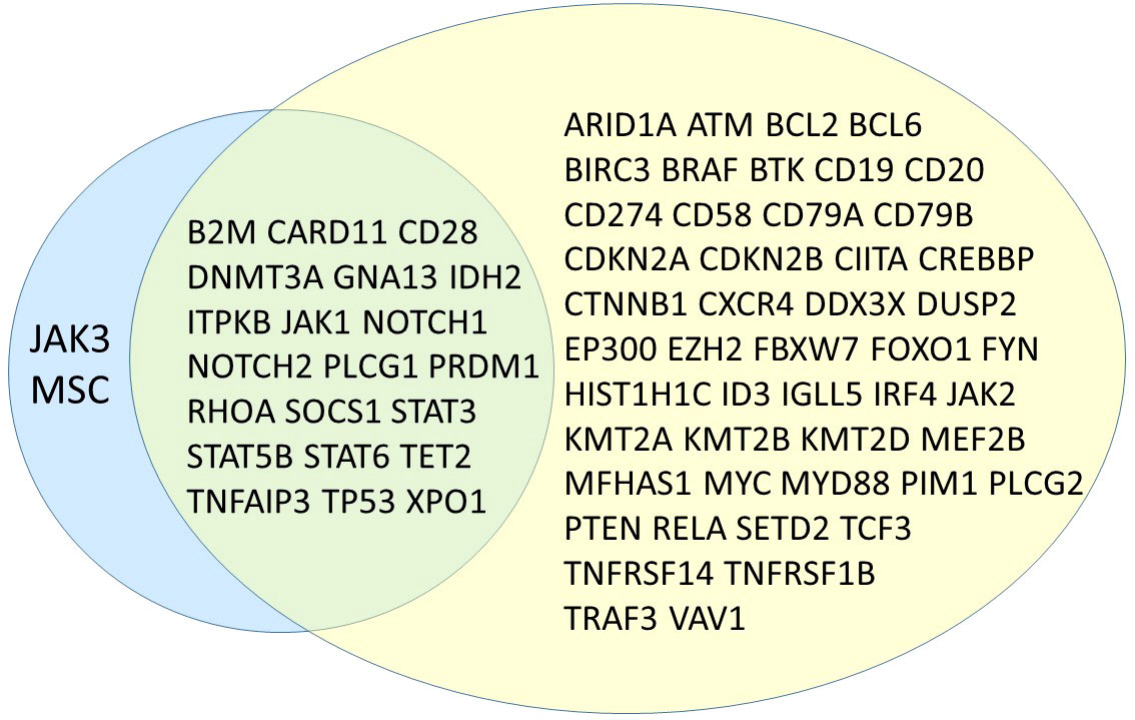
Venn diagram showing overlapping genes between the proposed panel in this study (left, blue) and the one presented by Syrykh et al. (right, yellow) (10).

We found one or more likely oncogenic or oncogenic variants in 89 samples and 6 samples with only VUS, which is higher than the number reported by Pillonel et al. and Davis et al., but lower than the mutation rate declared by Syrykh et al. However, the French group has used capture-based library generation and a different bioinformatics pipeline for variant calling, which might explain the differences with both our results and those of previous studies. A major source of variance is of course panel design itself, which in our case was focused on the inclusion of genes known to be mutated mostly in T-cell lymphomas, whereas the panels reported in the other publications contained also genes known to be mutated in both B- and T-cell neoplasias. **Figure 3** visualizes the overlap between our panel and the one designed by the French group. The comparison of both panels shows that most genes missing in our panel are indeed B-cell lymphoma associated genes, being diagnostically and biologically less relevant in T-cell lymphomas. We deliberately chose not to include large tumor-suppressor genes (except for *TET2*), especially if they rarely occurred as isolated mutations, since they would dramatically increase the panel size. On the other hand, we included part of genes which are frequently mutated in Hodgkin lymphomas (*STAT6*, *B2M*), since in our experience the differential diagnosis between a T-cell lymphoma with Hodgkin- and Reed-Sternberg-like cells and true classical Hodgkin lymphoma can be challenging and therapeutically relevant. The efficiency of our panel is shown in the percentage of mutated genes (21/23; 91%), compared with 39/69 (57%) in the panel designed by the French authors. This is also reflected in the high number of detected variants that could be classified as biologically relevant mutations (85%, 212/249).

As already shown in the results section, our success in detecting mutations was entity-dependent, with a very high efficiency for the TFH-cell lymphoma group (nearly 91% detection rate). Otherwise, we had only a limited success with PTCL, NOS cases (about 57% detection rate), due to the more complex genetic landscape that is not reflected in our simple design. Our finding confirms previously described data in the literature, including that *RHOA* and *IDH2* mutations appear to be highly specific for TFH-cell lymphomas, since we found no mutations in any of the 21 tested PTCL, NOS or in any other entity).

Interestingly, sometimes the panel results suggested new approaches to old problems. For instance, ALCL showed no mutations in *TET2* and only one (1/12, 8%) mutation in *DNMT3A*, a finding that might help in the differential diagnosis between this entity and cases of PTCL, NOS, where *TET2* mutations were relatively frequent (6/21, 29%). In some cases, genetic data might be a surrogate for other analyses. As an example, it was previously published, that mutations in *MSC* are specific for ALCL cases bearing a DUSP22 translocation (19); although not perfectly sensitive (only 2/3 cases with the translocation showed a *MSC* mutation) the detection of this mutation might warrant the performance of an appropriate FISH test.

The correlation of our sequencing data with standard clonality detection showed that the latter still has a superior sensitivity (as expected from the biology of T-cells). We detected clonally rearranged T-cell receptor genes in 75/78 (96%) tested samples, among positive cases, 57 (73%) had at least one mutation, while 18 cases with no genetic alteration showed rearranged T-cell-receptor gene fragments. GeneScan analysis confirms itself as a very sensitive technique, which will probably continue to be performed; however, its relative lack of specificity (being positive also in some non-neoplastic conditions) supports the use of a more specific molecular technology to corroborate a lymphoma diagnosis.

We analyzed our cases without selecting for specific entities, but we might have a selection bias since we receive external cases for a second opinion. In most cases, the panel results were only of confirmatory nature, adding further objective data to the results of morphology and immunophenotyping. In some cases, however, the results helped in the differential diagnosis or confirmed the neoplastic nature of a minor component, which was suspected to be neoplastic. Emblematic cases, for example, were cases of T-cell lymphomas in which the associated EBV-positive B-cell proliferation was exceptionally dominant, obscuring the neoplastic T-cell component and making it difficult to assess both morphologically and immunophenotypically; in this case the combination of clonality studies and panel sequencing eventually provided evidence of T-cell neoplasia (also confirmed in a later biopsy, not included in this series). In one case of NLPHL with slightly atypical and expanded intranodular T-cells, the detection of a classic *RHOA* and *TET2* mutation provided definitive evidence of an associated T-cell lymphoma (which could be classified as nodal T-cell lymphoma with TFH-phenotype). In other cases with only minimal lymphomatous infiltration or an atypical clinical presentation (e.g., isolated lymphadenopathy, young age) and considering the clinical consequence, we felt more comfortable having additional objective data to support the diagnosis of malignant lymphoma. In addition, we had several clinical requests for detection of mutations, particularly for *TET2*, *STAT3, STAT5B*, *IDH2*, *DNMT3A*, *RHOA* (10 cases), as cases with *TET2* mutations may correlate with response to therapy with azacitidine (20–23).

In the context of low variant allele frequency in genes which are strongly correlated to clonal hematopoiesis (CH) like *TET2* or *DNMT3A* it is difficult (if not impossible) to certainly attribute the mutation to the lymphoma cells; in this case, we normally add a comment highlighting this difficulty and recommending further studies (on peripheral blood or bone marrow). In our opinion, it is important to highlight that no single element is *per se* completely diagnostic, and in case of doubt a re-biopsy might be discussed. The relationship between CH and T-cell lymphomas is biologically relevant and warrants further investigation, since both conditions probably derive from a common mutated progenitor and significant clinical correlations start to emerge (24).

In conclusion, we report our experience with a small and efficient targeted NGS panel that could be a powerful tool for the diagnosis of selected cases or a useful diagnostic aid for specific predictive purposes. Our panel could be easily reproduced and applied in routine molecular diagnostics at a single samples level and provide reliable results in a relatively short time. Moreover, its application could be a useful complement to classical clonality detection.

## Data availability statement

The datasets presented in this study can be found in online repositories. The names of the repository/repositories and accession number(s) can be found below: NCBI, PRJNA992223.

## Ethics statement

The studies involving humans were approved by Local ethics committee University of Würzburg. The studies were conducted in accordance with the local legislation and institutional requirements. The ethics committee/institutional review board waived the requirement of written informed consent for participation from the participants or the participants’ legal guardians/next of kin because use of left-over and anonymized material.

## Author contributions

AZ, AR and JB contributed to conception and design of the study. AZ, AR, EG-H, IA, GO, and KSK reviewed the samples. JB and AZ designed the panel, interpreted the data and wrote the first draft of the manuscript. JB and KM supervised the panel implementation and the lab work. JB analyzed the mutational data and classified the variants. JB, AZ, SB, and KM analyzed the clonality data. JB submitted the final draft of the manuscript. All authors contributed to manuscript revision, read, and approved the submitted version. All authors contributed to the article and approved the submitted version.
